# LPS-stimulated microglial cells promote ganglion cell death in organotypic cultures of quail embryo retina

**DOI:** 10.3389/fncel.2023.1120400

**Published:** 2023-03-15

**Authors:** Ana Sierra-Martín, Julio Navascués, Veronika E. Neubrand, M. Rosario Sepúlveda, David Martín-Oliva, Miguel A. Cuadros, José L. Marín-Teva

**Affiliations:** Department of Cell Biology, Faculty of Sciences, University of Granada, Granada, Spain

**Keywords:** microglia, retina, quail, LPS-stimulation, iNOS, nitric oxide, ganglion cell death, organotypic cultures

## Abstract

During development microglia colonize the central nervous system (CNS) and play an important role in programmed cell death, not only because of their ability to remove dead cells by phagocytosis, but also because they can promote the death of neuronal and glial cells. To study this process, we used as experimental systems the developing *in situ* quail embryo retina and organotypic cultures of quail embryo retina explants (QEREs). In both systems, immature microglia show an upregulation of certain inflammatory markers, e.g., inducible NO synthase (iNOS), and nitric oxide (NO) under basal conditions, which can be further enhanced with LPS-treatment. Hence, we investigated in the present study the role of microglia in promoting ganglion cell death during retinal development in QEREs. Results showed that LPS-stimulation of microglia in QEREs increases (i) the percentage of retinal cells with externalized phosphatidylserine, (ii) the frequency of phagocytic contacts between microglial and caspase-3-positive ganglion cells, (iii) cell death in the ganglion cell layer, and (iv) microglial production of reactive oxygen/nitrogen species, such as NO. Furthermore, iNOS inhibition by L-NMMA decreases cell death of ganglion cells and increases the number of ganglion cells in LPS-treated QEREs. These data demonstrate that LPS-stimulated microglia induce ganglion cell death in cultured QEREs by a NO-dependent mechanism. The fact that phagocytic contacts between microglial and caspase-3-positive ganglion cells increase suggests that this cell death might be mediated by microglial engulfment, although a phagocytosis-independent mechanism cannot be excluded.

## Introduction

Microglia are the resident macrophages of the central nervous system (CNS). They originate from yolk sac-derived precursors that colonize the brain rudiment at very early stages of embryogenesis and are maintained by proliferation throughout the entire life of the organism, as occurs in mammals, or are replaced by hematopoietic precursors of intraembryonic origin, as occurs in other animal species such as zebrafish and chicken (Gomez Perdiguero et al., 2013; Ginhoux and Prinz, 2015; Askew and Gomez-Nicola, 2017; Cuadros et al., 2022). Microglial colonization of the nervous tissue and cell death coincides with cell death in different parts of the developing vertebrate CNS (Perry et al., 1985; Ashwell, 1990, 1991; Ferrer et al., 1990; Dalmau et al., 1998; Peri and Nusslein-Volhard, 2008; Calderó et al., 2009; Rigato et al., 2011; Swinnen et al., 2013; Casano et al., 2016), including the retina (Hume et al., 1983; Ashwell, 1989; Egensperger et al., 1996; Marín-Teva et al., 1999; Francisco-Morcillo et al., 2014). This coincidence has been mainly related to the well-known role of microglia as professional phagocytes, whose phagocytic potential ensures the clearance of cell corpses (efferocytosis) in cell death regions (reviewed in Eyo and Dailey, 2013; Sierra et al., 2013; Marín-Teva et al., 2014; Mosser et al., 2017; Marquez-Ropero et al., 2020; VanRyzin, 2021).

In several parts of the developing CNS, evidence for a role of microglia in promoting neuronal and glial cell death has been provided (reviewed in Marín-Teva et al., 2014; Casano and Peri, 2015; Mosser et al., 2017; VanRyzin, 2021; Irfan et al., 2022), which can be mediated by different mechanisms including both engulfment-unrelated and engulfment-promoted cell death (EPCD). The latter one has been observed in the embryonic mouse retina (Anderson et al., 2019), postnatal mouse cerebellum (Marín-Teva et al., 2004; Li et al., 2019), hippocampus (Wakselman et al., 2008), amygdala (VanRyzin et al., 2019), *corpus callosum* (Nemes-Baran et al., 2020), and the developing cerebral cortex of prenatal and postnatal macaques and rats (Cunningham et al., 2013). EPCD appears to be an evolutionarily conserved developmental mechanism because, in addition to the mammalian CNS, it has also been demonstrated to occur in the nematode *Caenorhabditis elegans* (Hoeppner et al., 2001; Reddien et al., 2001; Johnsen and Horvitz, 2016) and in the fruit fly *Drosophila melanogaster* (Hakim-Mishnaevski et al., 2019). Microglial EPCD may occur during CNS development without apparent prior activation of apoptosis, a mechanism that has been termed “primary phagocytosis” or “phagoptosis” (reviewed in Brown and Neher, 2012, 2014; Vilalta and Brown, 2018), by which viable neurons or glia (or their precursors) can die as a result of the process of phagocytosis by microglial cells (Cunningham et al., 2013; Anderson et al., 2019; VanRyzin et al., 2019; Nemes-Baran et al., 2020). This mechanism is different from the so-called “secondary phagocytosis,” in which the main cause of cell death is apoptosis or necrosis and the dead or dying cells are phagocytosed to keep clean the CNS parenchyma. However, in many experimental situations in which microglial cells are observed engulfing cells in early stages of apoptosis, it is very difficult to differentiate whether the apoptotic process began earlier or, on the contrary, if it was a consequence of the process of phagocytosis. Thus, EPCD could take place after the induction of a reversible apoptotic program characterized by the activation of caspases (Marín-Teva et al., 2004; Wakselman et al., 2008; Li et al., 2019). As shown in *Caenorhabditis elegans* (Johnsen and Horvitz, 2016), cells which are going to die only would complete their apoptotic program after being phagocytosed, while non-phagocytosed cells might either die or recover despite of caspase activation (Mallat et al., 2005).

Interestingly, cells that are susceptible to die by EPCD would expose on their surface “eat-me” signals that can be recognized by different microglial receptors (reviewed in Brown and Neher, 2012; Sierra et al., 2013; Brown and Neher, 2014; Vilalta and Brown, 2018; Marquez-Ropero et al., 2020). The best-characterized “eat-me” signal in mammals is the phospholipid phosphatidylserine, which is externalized to the outer monolayer of the cell membrane from early stages of apoptosis or during cell stress (Arandjelovic and Ravichandran, 2015; Park and Kim, 2017; Nagata, 2018; Lemke, 2019). Microglial production of reactive oxygen/nitrogen species (RONS), such as O_2_^–^ (superoxide) anions and nitric oxide (NO) that can combine to originate the powerful oxidant peroxynitrite, can induce surface exposure of phosphatidylserine in stressed or dying cells which in turn elicit the formation of phagocytic contacts with microglial cells (Marín-Teva et al., 2004; Wakselman et al., 2008; Neher et al., 2011; Cunningham et al., 2013). Several *in vitro* studies using a variety of strategies also demonstrated the promotion of neuronal death by microglial phagocytosis (Neher et al., 2011, 2014; Neniskyte et al., 2011, 2014, 2016; Fricker et al., 2012a,b; Hornik et al., 2016). In addition to its contribution to apoptosis during CNS development, microglial phagoptosis of viable neurons has been involved in cellular loss observed in brain and retinal pathologies (Neher et al., 2013; Zhao et al., 2015; Luo et al., 2016; Brelstaff et al., 2018).

In previous studies, we described that microglial precursors enter the retina of the quail embryo from a central region occupied by the base of the pecten and the head of the optic nerve from the seventh day of incubation (E7) onward (Navascués et al., 1995; Marín-Teva et al., 1999). It is noteworthy that the chronotopographical pattern of tangential migration of microglial cells was highly coincident with the pattern of distribution of naturally occurring neuronal death in the ganglion cell layer (GCL). Furthermore, we previously used organotypic cultures of quail embryo retina explants (QEREs) to show that developing microglia exhibit an upregulation of inflammatory markers such as inducible nitric oxide synthase (iNOS) and NO (hereafter referred to basal RONS production). This basal RONS production was also demonstrated in the *in situ* quail retina during normal development (Sierra et al., 2014). We also demonstrated that the bacterial endotoxin lipopolysaccharide (LPS) induces an additional stimulation of microglia in QEREs, supported by the observation of morphological changes toward an amoeboid cell shape, enlargement of the lysosomal compartment, and iNOS upregulation and increased production of NO (Sierra et al., 2014). It is important to note that the promotion of neuronal death by EPCD depends on the functional state of microglial cells and iNOS upregulation, as shown under different experimental conditions both *in vitro* and *in vivo* (Neher et al., 2011, 2014; Fricker et al., 2012a,b; Cunningham et al., 2013; Hornik et al., 2016; Rodríguez et al., 2017).

Thus, in the present study we have investigated the putative role of microglia in promoting ganglion cell death during embryonic quail retinal development by using organotypic cultures of E8 QEREs. We show here that LPS-stimulation of microglia in cultured QEREs increases the percentage of cells with externalized phosphatidylserine, the frequency of phagocytic contacts between microglial and caspase-3-positive ganglion cells, microglial production of NO and effectively promotes a decline in the ganglion cell population, with a proportion of this cell population being rescued after iNOS inhibition.

## Materials and methods

### Animals

Retinas from quail (*Coturnix coturnix japonica*) embryos at 8 days of incubation (E8) and E9 were used in this study. E8 central retina explants, which had a suitable number of microglial cells localized exclusively on the vitreal surface were chosen to analyze the functional state of microglia. E8 retina explants obtained from more dorsal regions that have not yet been colonized by microglial cells, have also been used for some experiments as explants without microglia. E8 *in situ* retinas were employed to analyze microglial contacts with dying cells at the starting situation of the experiments. E9 *in situ* retinas were used to compare cell viability between E8 cultured retinal explants and non-cultured retinas at an equivalent developmental age. Experimental procedures were approved by the Animal Experimentation Ethics Committee of the University of Granada, following the guidelines of the European Union Directive 2010/63/EU on the protection of animals used for scientific purposes.

### Organotypic cultures of E8 retina explants

Quail embryo retina explants (QEREs) from E8 were cultured *in vitro* on 30-mm Millicell CM culture plate inserts (Millipore, Bedford, MA, USA; pore size 0.4 mm), according to the method previously described (Stoppini et al., 1991) with some modifications (Carrasco et al., 2011). Briefly, retinas were dissected and kept in cold Gey’s balanced salt solution (Sigma, St. Louis, MO) supplemented with 5 mg/mL glucose (Sigma) and 50 IU-mg/mL penicillin-streptomycin (Invitrogen, Paisley, United Kingdom). After removing the retinal pigmented epithelium, square explants (3 mm x 3 mm) containing the dorsal part of base of the pecten and the optic nerve head area were isolated from the central region of each retina and then placed on Millicell inserts, with the vitreal surface downward. Subsequently, these Millicell inserts were put in six-well plates containing 1 mL/well culture medium composed of 50% basal medium with Earle’s salts, 25% Hank’s balanced salt solution, 25% horse serum, 1 mM L-glutamine, 10 IU-mg/mL penicillin-streptomycin (all purchased from Invitrogen), and 5 mg/mL glucose. E8 QEREs were then incubated at 37°C in a humidified atmosphere with 5% CO_2_ for 1 h *in vitro* (hiv) to 24 hiv (E8 + 1hiv to E8 + 24hiv, respectively).

### LPS-stimulation of microglial cells in cultured retina explants

Microglial cells were stimulated by treating E8 QEREs with 5 μg/mL LPS (Escherichia coli OB4:1111, Sigma). In each experiment, the explant obtained from the right eye was LPS-treated, while the explant obtained from the left retina of the same embryo was cultured without LPS as control.

### Antibodies

The QH1 monoclonal antibody (dilution 1:4, Developmental Studies Hybridoma Bank, University of Iowa, Iowa City, IA) labels all quail hemangioblastic cells except mature erythrocytes (Pardanaud et al., 1987), including immature, ramified, and reactive microglia (Cuadros et al., 1992). Because the quail retina is avascular, QH1 only labels microglia in retinal explants (Carrasco et al., 2011; Sierra et al., 2014). The 39.4D5 monoclonal antibody (dilution 1:100, Developmental Studies Hybridoma Bank, University of Iowa, Iowa City, IA) recognizes the transcription factor Islet 1 and labels most ganglion cells and other subsets of retinal neurons (Fischer et al., 2002; Halfter et al., 2005). In addition, the anti-caspase-3 polyclonal antibody (dilution 1:100, R&D Systems, Minneapolis, MN) was used as marker for apoptotic cells in embryo retina (Borsello et al., 2002). As secondary antibodies we used Alexa Fluor 488-conjugated goat anti-mouse IgG (dilution 1:1,000, Molecular Probes, Eugene, OR) and Cy3-conjugated goat anti-rabbit IgG (dilution 1:1,000, Healthcare Europe, Freiburg, Germany).

### Immunolabeling of whole-mounted and cryosectioned retinal explants

Double QH1/anti-caspase-3 immunolabeling was carried out on whole-mounted non-cultured retinas and cultured retina explants, which were fixed with 4% paraformaldehyde in 0.1 M phosphate buffer for 1 h and permeabilized with phosphate buffered saline (PBS) containing 1% Triton X-100 (PBS-1%T) for 4 h. They were then rinsed with PBS containing 0.1% Triton X-100 (PBS-T) and incubated with 1% normal goat serum (NGS) in PBS, containing 1% bovine serum albumin (BSA) and 0.25% Triton X-100 (PBS-BSA-T) for 1h at room temperature. After incubation with a mixture of the primary antibodies QH1 and anti-caspase-3 in PBS-BSA-T for 48 h at 4°C, they were rinsed with PBS-T and incubated with a mixture of the secondary antibodies in PBS-BSA-T for 4 h at room temperature. After rinsing with PBS-T, nuclei of whole-mounted retina explants were stained with the nuclear dye Hoechst 33342 (Sigma) diluted at 10 μg/ml in PBS for 5 min. Then, the explants were mounted with the vitreal side upward on coverslips with Fluoromount G (Southern Biotech, Birmingham, AL).

Cross cryosections of non-cultured retinas and cultured retina explants were also used for double QH1/anti-caspase-3 immunolabeling. Retinas were fixed for 1 h at 4 °C with 4% paraformaldehyde in 0.1 M phosphate buffer, thoroughly rinsed with PBS-T, cryoprotected overnight at 4°C with 20% sucrose in PBS-T and introduced into 7.5% gelatin and 20% sucrose in PBS-T. Solidified blocks containing the specimens were then embedded in OCT compound, frozen in isopentane cooled with liquid nitrogen, and stored at -40°C before sectioning on a Leica CM1850 cryostat. Cryosections of 15 μm-thick were collected on Superfrost slides (Menzel-Glasser, Braunschweig, Germany), hydrated in PBS, and labeled by double QH1/anti-caspase-3 immunocytochemistry. The immunocytochemical procedure for cryosections was similar to the one described above for wholemounts with the following differences: cryosections were not permeabilized, incubation time with secondary antibodies was reduced to 2.5 h and Hoechst 33342 incubation was reduced to 3 min.

For single immunolabeling, cryosections of retinal explants were incubated with the monoclonal antibody 39.4D5 (anti-Islet 1) overnight, followed by nuclei staining with Hoechst 33342. Subsequently, the secondary antibody Alexa Fluor 488-conjugated goat anti-mouse IgG diluted 1:1000 in PBS-BSA-T was added for 2.5 h.

### Determination of cell viability

Cell viability was determined by flow cytometry of cell suspensions obtained by dissociation of retinal explants, in accordance with the technique described in Jones and Senft (1985), using fluorescein diacetate (FDA, Sigma) and propidium iodide (PI, Sigma). Non-fluorescent FDA is a membrane-permeable esterase substrate that is cleaved by the esterase activity of living cells and gives rise to fluorescein, which emits green fluorescence and is retained within viable cells. On the contrary, PI enters cells and binds to cell DNA, producing a red fluorescence, only when the integrity of the cell membrane is compromised, therefore marking dead or dying cells.

Briefly, retinal explants were incubated for 10 min in the dark with 10 mg/ml PI and 15 mg/ml FDA. Then, they were dissociated using a Dounce homogenizer (Pobel, Madrid, Spain), and the resulting suspension was analyzed by flow cytometry using the FACS Vantage flow cytometer (Beckton Dickinson, Franklin Lakes, NJ) with band pass filters of 530 nm (for FDA fluorescence) and 580 nm (for PI fluorescence). Cell viability was determined in each sample as the percentage of FDA-positive cells from the total cell number (FDA-positive and PI-positive cells).

### Quantification of annexin V-positive cells

Retinal explants were incubated with EGFP-Annexin V (BioVision, dilution 1:500) to label dying or stressed cells and Alexa Fluor 594-conjugated QH1 antibody (AF594-QH1, 0.4 mg/ml) for 1 h. The conjugation of QH1 antibody with Alexa Fluor 594 was performed as previously reported (Carrasco et al., 2011). Then, explants were dissociated as described above and the resulting cell suspension was analyzed by flow cytometry using the FACS Canto II cytometer (Becton-Dickinson). To analyze only dead neurons but not microglial cells, AF594-QH1 labeled cells and double AF594-QH1/Annexin V labeled cells were discarded from the analysis.

### TUNEL staining

Localization of dying cells was assessed with the TUNEL technique in both whole-mounts and cryosections of LPS-treated and non-treated E8 + 24hiv retina explants.

Whole-mounted explants were fixed with 4% paraformaldehyde in 0.1 M PBS overnight and permeabilized with PBS-1%T for 3 h at 4°C. After rinsing with PBS-0.1%T, explants were treated in a solution with Proteinase k (Sigma) in Tris-HCl buffer (20 μg/ml) to inactivate endogenous nucleases. Subsequently, a second fixation with 4% paraformaldehyde in 0.1 M phosphate buffer for an additional 3 h was performed. Following an incubation of 2 h at 37°C with TdT buffer (Promega), containing 8 nmol/ml of biotin-16-dUTP (Roche Diagnostics) and 20 U/ml of TdT (Promega), explants were rinsed and then incubated with AF488-conjugated streptavidin (Molecular Probes) in PBS-T for 45 min. After washing, explants were stained with Hoechst 33342 to label nuclei and subsequently mounted with Fluoromount G.

A similar procedure was followed for cryosections. After hydration and permeabilization, sections were treated with a solution containing 4 nmol/ml of biotin-16-dUTP (Roche Diagnostics) and 10 U/ml of TdT (Promega) in TdT buffer for 1 h at 37°C. After rinsing, cryosections were incubated with AF488-conjugated streptavidin, nuclei were stained with Hoechst 33342 and cryosections were mounted with Fluoromount.

To quantify the relative area occupied by TUNEL profiles in the ganglion cell layer (GCL), three square microscopic fields (250 μm × 250 μm) were selected in each explant, in which the percentage of pixels occupied by TUNEL-positive profiles in each field was determined using Image Tool 2.0 software (University of Texas Health Science Center, San Antonio, TX).

### Quantitative analysis of Islet 1-positive cells

Explants were fixed with 4% paraformaldehyde in 0.1 M phosphate buffer for 10 min and immunolabeled with a solution containing the monoclonal antibody 39.4D5 (anti-Islet 1, dilution 1:100) and 1% NGS in PBS-BSA-T at 4°C overnight. After rinsing with PBS-0.1%T, explants were incubated with the secondary antibody AF488-conjugated goat anti-mouse IgG diluted 1:1000 in PBS-BSA-T. Then, explants were dissociated and analyzed by the FACS Canto II cytometer.

Cryosections of retina explants were used to quantify the number of Islet 1-positive cells in the GCL. Three cryosections were analyzed in each explant: one of the dorsal zone, one of the central zone and one of the ventral zone, using a Zeiss Axiophot microscope (Zeiss, Oberkochen, Germany). In each zone, micrographs (250 μm wide) were taken using a Zeiss AxioCam digital camera and Islet 1-positive nuclei were quantified in the GCL, determining the number of Islet 1-positive ganglion cells per 250 μm of GCL length.

### Cell-to-cell contacts between microglia and caspase-3 positive ganglion cells

Cell-to-cell contacts were analyzed by double staining with anti-caspase-3 and QH1 antibodies, and Hoechst 33342 for nuclei detection. Maximum projections of confocal sections taken with a 63x objective in three different regions (dorsal, nasal and temporal) in each explant were examined, using a Leica TCS-SP5 confocal microscope (Leica, Wetzlar, Germany). Caspase-3-positive ganglion cells in contact with microglia and caspase-3-positive ganglion cells without cell-to-cell contacts were quantified. To detect them, an analysis of all optical sections integrated in maximum projections was performed. Finally, average percentages of caspase-3-positive ganglion cells in contact with microglia, microglial cell densities and caspase-3-positive ganglion cell densities were determined by analyzing microscopic fields of 500 μm × 500 μm in three different zones (dorsal, nasal and temporal) of 10 explants treated with LPS and 10 untreated controls.

### Microglial ROS detection

Explants were labeled for 1 h at 37°C with the fluorescent dye carboxy-2′7′-dichlorodihydrofluorescein diacetate (H_2_DCFDA, Molecular Probes, 20 μM), which is converted to the green-fluorescent form carboxy-DCF after ROS oxidation (Gabriel et al., 1997; Jakubowski and Bartosz, 2000), and AF594-QH1 (0.4 μg/ml) as microglial marker. After rinsing with PBS and fixation with 4% paraformaldehyde in 0.1 M phosphate buffer, mounted explants were photographed obtaining maximal projections with a 63x objective using a Leica TCS-SP5 confocal microscope (Leica, Wetzlar, Germany).

Some of the explants mentioned above were used to analyze the percentage of microglial cells producing ROS by flow cytometry. Briefly, suspensions obtained from double carboxy-DCF/AF594-QH1 labeled retina explants were analyzed by the FACS Canto II cytometer, obtaining the percentage of DCF-positive microglia of total microglia population.

In addition, superoxide anion production by microglia was analyzed by flow cytometry, using retina explants labeled for 1 h at 37°C with dihydroethidium (DHE, Molecular Probes, 5 μM) and AF594-QH1 (0.4 μg/ml). DHE reacts with superoxide anions generating oxidized ethidium that is accumulated in the cellular nucleus displaying red fluorescence. The percentage of superoxide anions-producing microglial cells was determined in the same manner as described above for the DCF-dye.

### iNOS inhibition through L-NMMA treatment

The iNOS inhibitor NG-Monomethyl-L-arginine, Monoacetate (L-NMMA, Calbiochem) at a concentration of 1 nM, and LPS at a concentration of 5 μg/mL were added to the culture medium. LPS-treated explants were used as controls. The explants were cultured for 12 hiv or 24 hiv depending on the experimental setup.

### Statistical analysis

Data are reported as means ± standard error of the mean (SEM). Statistical differences were determined with the Student’s t-test and by one-way analysis of variance (ANOVA) followed by Tukey test for multiple comparisons. All analyses were performed using IBM SPSS statistics software version 21.0.0 for Windows (Chicago, IL, USA). Differences were considered significant at *P* < 0.05.

## Results

### Immature microglia in E8 quail embryo retina explants (QEREs) react to *in vitro* LPS treatment

**FIGURE 1:**
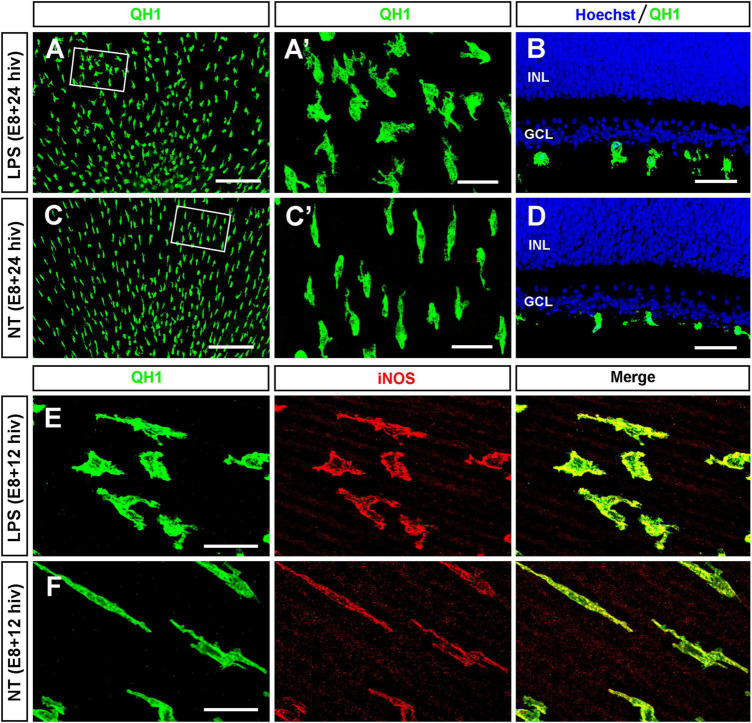
LPS-treatment of E8 retina explants induces changes in morphology and iNOS expression in immature microglia. **(A–D)** Representative images of QH1-immunolabeled microglia (green) in whole-mounts **(A,C)** and cross cryosections **(B,D)** of LPS-treated [LPS; **(A,B)**] and non-treated [NT; **(C,D)**] retina explants from E8 quail embryos cultured for 24 h (E8 + 24 hiv). Hoechst nuclear staining (blue) was used to visualize the retinal layers in cryosections (INL: inner nuclear layer; GCL: ganglion cell layer). The boxed areas in panels **(A,C)** are shown at a higher magnification in panels **(A’,C’)**, respectively. Note morphological changes of microglial cells in LPS-treated explants **(A,A’,B)** compared with those in the non-treated explants **(C,C’,D)**. **(E,F)** Representative confocal images of QH1 (green) and anti-iNOS (red) double immunostained microglial cells in LPS-treated [LPS; **(E)**] and non-treated [NT; **(F)**] E8 + 12 hiv retina explants. Green, red, and merged channels are shown in the left (QH1), medium (iNOS), and right (Merge) panels, respectively. In addition to that, morphological changes similar to those found in E8 + 24 hiv retina explants, the iNOS labeling of microglia is higher in LPS-treated versus non-treated explants. Scale bars: 200 μm in panels **(A,C)**; 40 μm in panels **(A’,B,C’,D)**; 25 μm in panels **(E,F)**.

First, we confirmed that microglia were able to respond to LPS treatment of E8 QEREs cultured for either 12 or 24 h *in vitro* (E8 + 12 hiv and E8 + 24 hiv) under our experimental conditions (Figure 1), as previously shown (Sierra et al., 2014). We used the developmental age of E8 because microglial colonization and ganglion cell death begin at E7 in the quail embryo retina (Marín-Teva et al., 1999). Hence, at E8 a considerable, although still limited number of microglial cells has already entered the retina and show their typical central-to-peripheral polarized migratory morphology. The bacterial endotoxin LPS was chosen because it is a pro-inflammatory stimulus that induces an inflammatory response in immature microglia in the developing brain (Hristova et al., 2010; Li et al., 2010; Cunningham et al., 2013; Manivannan et al., 2013). Importantly, we previously demonstrated that microglia in E8 QEREs became reactive upon LPS treatment for 24 hiv, showing a more rounded and less elongated and polarized morphology, that was associated to an upregulated iNOS expression and a concomitant production of NO (Sierra et al., 2014) as pro-inflammatory markers. In the present study, we show that upregulated iNOS expression in microglia was found already after 12 hiv LPS treatment (Figures 1E, F). Since microglia exhibit a basal RONS production in the developing CNS (Crain et al., 2013; Sierra et al., 2014) and microglial EPCD is dependent on microglial inflammatory responses (Cunningham et al., 2013; Neher et al., 2014; Neniskyte et al., 2014; Hornik et al., 2016; Rodríguez et al., 2017), we explored the hypothesis that modifying the functional state of microglia by LPS-stimulation was related to the viability of retinal ganglion cells.

### LPS treatment decreases cell viability and increases cell death in cultured QEREs

**FIGURE 2:**
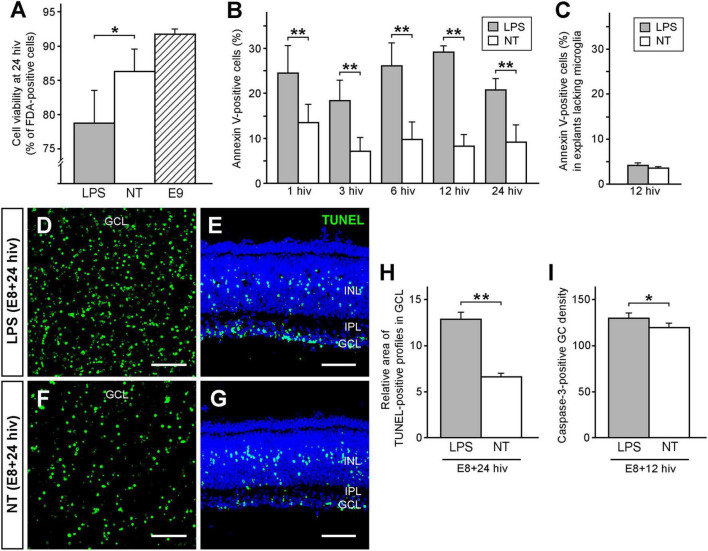
LPS treatment decreases cell viability and increases cell death in retina explants prepared from quail embryos cultured for 24 h *in vitro* (E8 + 24 hiv). **(A)** Cell viability in LPS-treated (LPS, grey bar) and non-treated (NT, white bar) E8 + 24 hiv retina explants compared with that in non-cultured E9 retina explants (E9, striped bar). Cell viability is expressed as the percentage of fluorescein diacetate (FDA)-positive cells from the whole cell population, which was obtained by flow cytometry analysis of retina explant cell suspensions treated with FDA, that labels viable cells, and propidium iodide, that stains dead cells. Data are expressed as means ± SEM (*n* = 9 for each condition). The percentage of viable cells is significantly lower in LPS-treated E8 + 24 hiv retina explants versus non-treated explants, while it is not significantly different between non-treated explants and non-cultured E9 retina explants (*P < 0.05, one-way ANOVA followed by Tukey test for multiple comparisons). **(B)** Percentages of annexin V-positive cells in E8 retina explants cultured for 1, 3, 6, 12, and 24 hiv in the presence (grey bars) or the absence (white bars) of LPS, as determined by flow cytometry analysis of cell suspensions from retina explants stained with EGFP-conjugated annexin V. Data are expressed as means ± SEM (*n* = 6 for each condition). The percentages of annexin V-positive cells are significantly higher in LPS-treated than in non-treated explants at all *in vitro* time points (**P < 0.01, one-way ANOVA followed by Tukey test for multiple comparisons). **(C)** Percentages of annexin V-positive cells in E8 retina explants lacking microglia cultured for 12 hiv in the presence (grey bars) or the absence (white bars) of LPS, as determined by flow cytometry analysis of cell suspensions from retina explants stained with EGFP-conjugated annexin V. Data are expressed as means ± SEM (*n* = 6 for each condition). Note that there are no significant differences between the percentages of annexin V-positive cells in LPS-treated and non-treated explants (*P* = 0.14, Student’s t-test). **(D–G)** Representative confocal images of TUNEL-stained (green) whole-mounts **(D,F)** and cross sections **(E,G)** from LPS-treated [LPS; **(D,E)**] and non-treated [NT; **(F,G)**] E8 + 24 hiv retina explants. Images of whole-mounted explants are maximum-intensity projections from Z stacks of confocal optical sections throughout the thickness of the ganglion cell layer (GCL). Explant cross-sections were stained with Hoechst 33342 (blue) to visualize the retinal layers (INL: inner nuclear layer; IPL: inner plexiform layer). Note that TUNEL-positive profiles in the GCL are more abundant in LPS-treated **(D,E)** than non-treated **(F,G)** explants. Scale bars: 50 μm. **(H)** Relative areas of TUNEL-positive profiles in the GCL of LPS-treated (LPS, grey bar) and non-treated (NT, white bar) E8 + 24 hiv retina explants. Bars represent percentages of TUNEL-positive pixels in the GCL of whole-mounted explants as measured on three microscopic fields of 250 μm × 250 μm per explant. Data are represented as means ± SEM (*n* = 17 for each condition). The relative area of TUNEL-positive profiles is significantly higher in LPS-treated versus non-treated explants (**P < 0.01, Student’s t-test). **(I)** Caspase-3-positive ganglion cell densities in LPS-treated (LPS, grey bar) and non-treated (NT, white bar) E8 + 12 hiv retina explants. Data are presented as means (±SEM) of caspase-3-positive ganglion cells per mm^2^ obtained from counts in three square fields of 500 μm × 500 μm per whole-mounted retina explant (*n* = 6 for each condition). The density of caspase-3-positive ganglion cells is significantly higher in LPS-treated than in non-treated explants (*P < 0.05, Student’s t-test).

Flow cytometry analysis revealed that the percentage of viable cells in LPS-treated E8 + 24 hiv QEREs was significantly lower (78.7% ± 4.8) than in non-treated control QEREs (86.3% ± 3.3), showing that LPS had a negative effect on viability of developing retinal cells (Figure 2A). E9 QEREs were used to compare cell viability with non-treated E8 + 24 hiv QEREs, and no significant differences in cell viability were detected (Figure 2A), revealing that the culture of QEREs for 24 hiv did not significantly affect cell viability.

To analyze this negative effect of LPS on cell viability in E8-cultured QEREs we then used different experimental approaches that complement each other:

First, we determined the percentage of dying or stressed cells by flow cytometry on cell suspensions of QEREs. Thus, cells with externalized phosphatidylserine were labeled with enhanced green fluorescent protein-conjugated annexin V (EGFP-annexin V). Percentages of annexin V-positive cells in E8 QEREs cultured for 1, 3, 6, 12, and 24 hiv were significantly higher in LPS-treated QEREs than in control QEREs for all tested time points with a maximal difference observed at 12 hiv (Figure 2B). To verify that microglia are the responsible cell type for this phosphatidylserine externalization on cells in LPS-stimulated E8 QEREs, we prepared E8 retinal explants from a more dorsal region of the retina which microglial cells have not yet reached at this developmental stage. Thus, they can be considered as explants lacking microglia (Figure 2C). Remarkably, we did not observe any significant differences between these explants treated with LPS and controls at 12 hiv, demonstrating LPS-stimulated microglia are responsible for the phosphatidylserine externalization detected in the experiments described above. Note that the values of annexin V-positive cells obtained in these explants without microglia in both experimental conditions are a little bit lower than those in Figure 2B at 12 hiv, probably because it is an adjacent region located more dorsally in which cell death is delayed, as previously shown by Marín-Teva et al. (1999) in distribution maps of microglia and neuronal death in the embryonic quail retina.

Second, based on these results and given that the duration of cell death processes requires several hours, the time point of 24 hiv was selected to analyze the extent of dying cells in final stages in TUNEL assays. The TUNEL-positive profiles in the ganglion cell layer (GCL) were apparently more abundant in LPS-treated E8 + 24hiv cultures than in non-treated cultures, as seen in TUNEL-labeled whole-mounts and cross sections of QEREs (Figures 2D–G). Quantitative analysis in the GCL of whole-mounted QEREs confirmed these findings, as the relative area of TUNEL-positive profiles (percentage of TUNEL-positive pixels per unit area) in LPS-treated QEREs was 12.8% ± 0.7) compared with non-treated QEREs (6.6% ± 0.4) (Figure 2H).

Finally, considering that caspase-3 activation occurs in earlier stages of cell death, the time point of 12 hiv was selected to analyze the expression of activated-caspase-3 in GCL. The density of caspase-3-positive ganglion cells (number of caspase-3-positive ganglion cells per mm^2^) was significantly higher in LPS-treated QEREs (129.55 ± 7.79) than in non-treated cultures (119.55 ± 4.80) (Figure 2I).

All these results demonstrated that ganglion cell death was increased in response to LPS treatment and that the presence of microglia in E8 QEREs was necessary for this process.

### LPS treatment induces a decline of the ganglion cell population in E8 + 24 hiv QEREs

**FIGURE 3:**
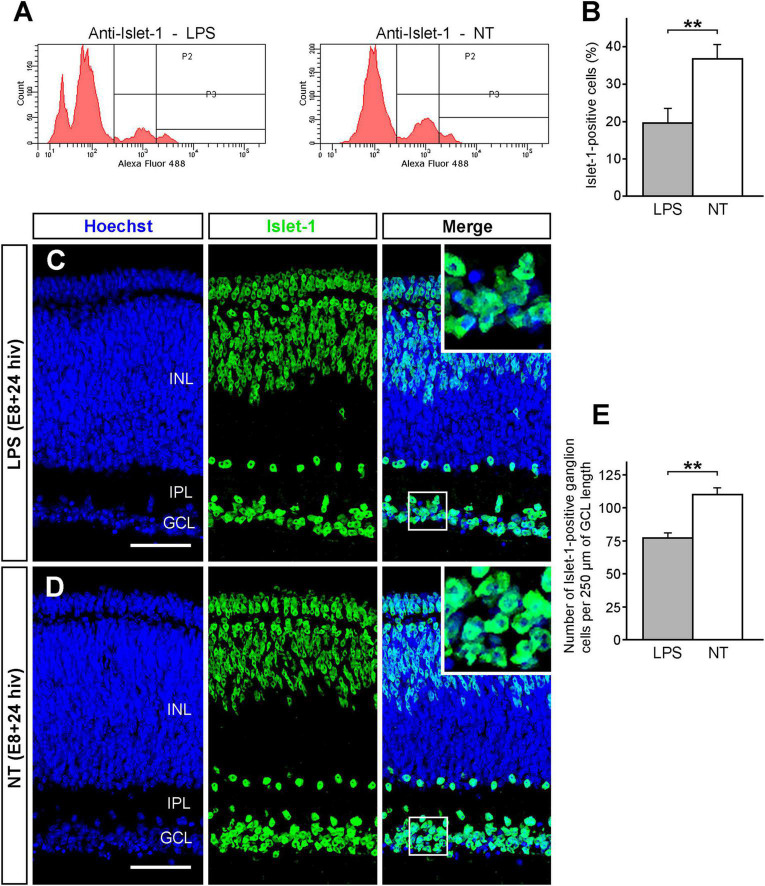
Decrease of the ganglion cell population after LPS treatment. The Islet 1-positive ganglion cell population significantly declines after LPS treatment in retina explants from E8 quail embryos cultured for 24 h *in vitro* (E8 + 24 hiv). **(A)** Representative histograms showing flow cytometry analysis of cell suspensions from LPS-treated (LPS) and non-treated (NT) E8 + 24 hiv retina explants immunolabeled with the anti-Islet 1 antibody and revealed with Alexa Fluor-488-conjugated anti-mouse IgG. In the two histograms, P2 and P3 represent the Islet 1-positive cell population that is distinctly lower in LPS-treated than non-treated explants. **(B)** Percentages (means ± SEM) of Islet 1-positive cells obtained in the flow cytometry analysis of cell suspensions from LPS-treated (LPS, grey bar) and non-treated (NT, white bar) retina explants (*n* = 8 for each condition). The Islet 1-positive cell percentage is significantly lower in LPS-treated versus non-treated explants (**P < 0.01, Student’s t-test). **(C,D)** Representative confocal images of anti-Islet 1 (green) immunolabeled cross-sections from LPS-treated [LPS; **(C)**] and non-treated [NT; **(D)**] E8 + 24 hiv retina explants. Nuclear stain with Hoechst 33342 (blue) was used to visualize the retinal layers (INL: inner nuclear layer; IPL: inner plexiform layer; GCL: ganglion cell layer). Blue, green and merged channels are shown in the left (Hoechst), medium (Islet 1) and right (Merge) panels, respectively. Insets in the right panels show higher magnifications of the boxed areas. The Islet 1-positive ganglion cells appear to be less numerous in LPS-treated explants **(C)** compared to non-treated ones **(D)**. Scale bars: 45 μm. **(E)** Quantification of Islet 1-positive cells in the GCL of LPS-treated (LPS, grey bar) and non-treated (NT, white bar) E8 + 24 hiv retina explants. The quantitative analysis was performed on three cross sections from dorsal, medial, and ventral parts of LPS-treated and non-treated explants (*n* = 8 for each condition). Data are expressed as means (±SEM) of numbers of Islet 1-positive cells per 250 μm of GCL length in each histological section. The number of Islet 1-positive ganglion cells is significantly lower in LPS-treated than in non-treated explants (**P < 0.01, Student’s t-test).

The transcription factor Islet 1 is expressed in different subpopulations of avian retinal neurons, including ganglion cells (Fischer et al., 2002; Halfter et al., 2005; Carrasco et al., 2011). Flow cytometry was used to assess the effect of the LPS treatment on Islet 1-positive retinal neurons in E8 + 24 hiv QEREs (Figure 3A). LPS treatment significantly decreased the percentage of Islet 1-positive cells from 36.8% (±3.8) in non-treated QEREs to 19.6% (± 4.1) in LPS-treated ones (Figure 3B). This drop in the Islet 1-positive cell percentage is compatible with a decrease in the number of ganglion cells. Indeed, the GCL thickness in LPS-treated QEREs seemed smaller than in non-treated QEREs (Figures 3C, D) and the quantification of ganglion cell densities on Islet 1 immunolabeled cross sections showed that the number of Islet 1-positive cells per 250 μm of GCL length decreased about 30% after LPS treatment (Figure 3E). These results demonstrated that LPS treatment of E8 + 24 hiv QEREs induced a significant reduction in the ganglion cell number that was in concordance with the increase in ganglion cell death, as described above.

### Phagocytic contacts frequency between microglial cells and caspase-3-positive ganglion cells is increased after LPS treatment in E8 + 12 hiv QEREs

**FIGURE 4:**
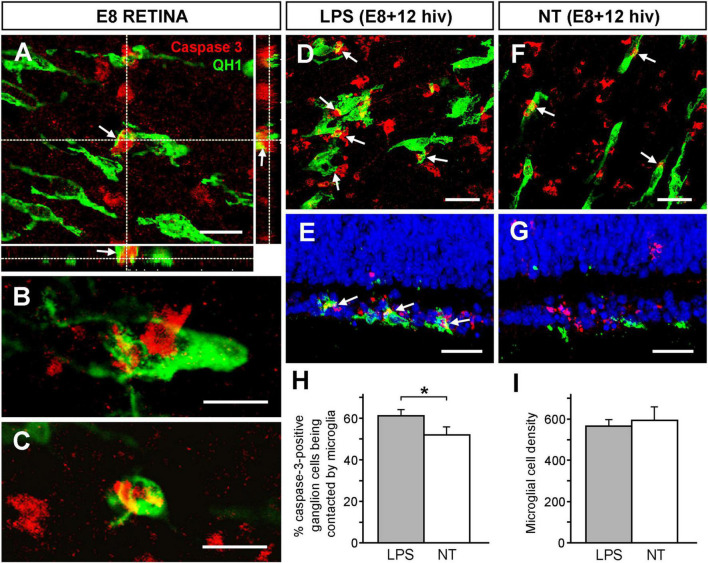
Increase of phagocytic contacts between microglial cells and caspase-3-positive ganglion cells. The frequency of phagocytic contacts between microglial cells and caspase-3-positive ganglion cells increases in response to LPS treatment in retina explants from E8 quail embryos cultured for 12 h *in vitro* (E8 + 12 hiv). **(A–C)** Confocal images of microglial cells forming close engulfing contacts with caspase-3-positive ganglion cells in whole-mounted non-cultured E8 quail embryo retina explants, (E8 RETINA) double immunolabeled with QH1 (green) and anti-caspase-3 (red). These images are maximum-intensity projections from Z stacks of confocal optical sections throughout the thickness of the ganglion cell layer (GCL). Panel **(A)** shows a process from a microglial cell that contacts and embraces a caspase-3-positive ganglion cell. The XZ and YZ orthogonal projections corroborate the close contact (arrows) between the microglial cell process and the ganglion cell. Scale bar: 15 μm. **(B)** Phagocytic contacts between a microglial cell soma and a caspase-3-positive ganglion cell are observed. **(C)** A caspase-3-positive ganglion cell completely engulfed by a microglial cell. Scale bars for panels **(B,C)**: 15 μm. **(D–G)** Representative confocal images of whole-mounts **(D,F)** and cross-sections **(E,G)** from LPS-treated [LPS; **(D,E)**] and non-treated [NT; **(F,G)**] E8 + 12 hiv retina explants double immunolabeled with QH1 (green) and anti-caspase-3 (red) showing phagocytic contacts (arrows) between microglial cells and caspase-3-positive ganglion cells. Confocal images of whole-mounted explants are optical sections obtained in the ganglion cell layer. Cell nuclei in cross-sections were stained with Hoechst 33342 (blue) to visualize the retinal layers. Phagocytic contacts between microglial cells and caspase-3-positive ganglion cells are more abundant in LPS-treated explants compared to non-treated ones. Scale bars: 25 μm. **(H)** Quantification of caspase-3-positive ganglion cells that are contacted by microglia in LPS-treated (grey bar) and non-treated (white bar) E8 + 12 hiv retina explants. Bars represent means (±SEM) of percentages obtained from counts in three different zones (dorsal, nasal and temporal) of whole-mounted explants (*n* = 6 for each condition). The percentage of caspase-3-positive ganglion cells being contacted by microglia is significantly higher in LPS-treated than in non-treated explants (*P < 0.05, Student’s t-test). **(I)** Microglial cell densities in LPS-treated (LPS, grey bar) and non-treated (NT, white bar) E8 + 12 hiv retina explants. Data are presented as means (±SEM) of microglial cells per mm^2^ obtained from counts in three square fields of 500 μm × 500 μm per whole-mounted retina explant (*n* = 10 for each condition). No significant differences are detected between LPS-treated and non-treated explants.

Considering that microglia establish close contacts with neurons before they are killed by EPCD (Marín-Teva et al., 2004; Gibbons and Dragunow, 2006; Wakselman et al., 2008; Virgone-Carlotta et al., 2013), the frequency of microglial phagocytic contacts with caspase-3-positive ganglion cells was assessed. These contacts were clearly identified in confocal optical sections of quail embryo *in situ* retinas at E8, a time point when intense programmed death of developing ganglion cells takes place (Marín-Teva et al., 1999). Microglial cell processes were visualized contacting and “hugging” caspase-3-positive ganglion cells, as evidenced by XZ and YZ orthogonal projections of confocal Z-stacks (Figure 4A). Phagocytic contacts were also observed between caspase-3-positive ganglion cell somas and microglial cell bodies (Figure 4B) and some caspase-3-positive ganglion cells were within microglial cells (Figure 4C).

The presence of phagocytic contacts between microglial cells and caspase-3-positive ganglion cells was analyzed on whole-mounts (Figures 4D, F) and cross sections (Figures 4E, G) of LPS-treated (Figures 4D, E) and non-treated (Figures 4F, G) E8 + 12 hiv QEREs. This time point of the *in vitro* culture (12 hiv) was selected because it presented the maximal difference in percentage of annexin V-positive cells between LPS-treated and non-treated E8 QEREs (see Figure 2B). Phagocytic contacts were evident in the GCL of LPS-treated and non-treated E8 + 12 hiv QEREs, although they were apparently more frequent in LPS-treated cultures (Figures 4D–G). Quantification in whole-mounted QEREs indeed demonstrated that the percentage of caspase-3-positive ganglion cells being contacted by microglia was significantly higher after LPS-treatment (Figure 4H). To test if a high number of contacts is due to an increase in the number of microglia, microglial cell densities were quantified, proving that the LPS treatment did not significantly increase the microglial cell density in E8 + 12 hiv cultures (Figure 4I). Therefore, we concluded that microglial cells were more engaged in establishing phagocytic contacts with caspase-3-positive ganglion cells after LPS-stimulation.

### LPS treatment induces in microglia a higher production of RONS but not of superoxide anions in E8 + 12 hiv QEREs

LPS-activated microglia increase the production of RONS, such as superoxide anions, NO and peroxynitrite, which can trigger neuronal degeneration, as demonstrated in *in vivo* and *in vitro* studies (Arimoto and Bing, 2003; Qin et al., 2004, 2013; Neher et al., 2011; di Penta et al., 2013). Based on this knowledge, we tested here whether RONS production by microglial cells is increased in LPS-treated E8 + 12 hiv QEREs when compared with non-treated ones.

**FIGURE 5:**
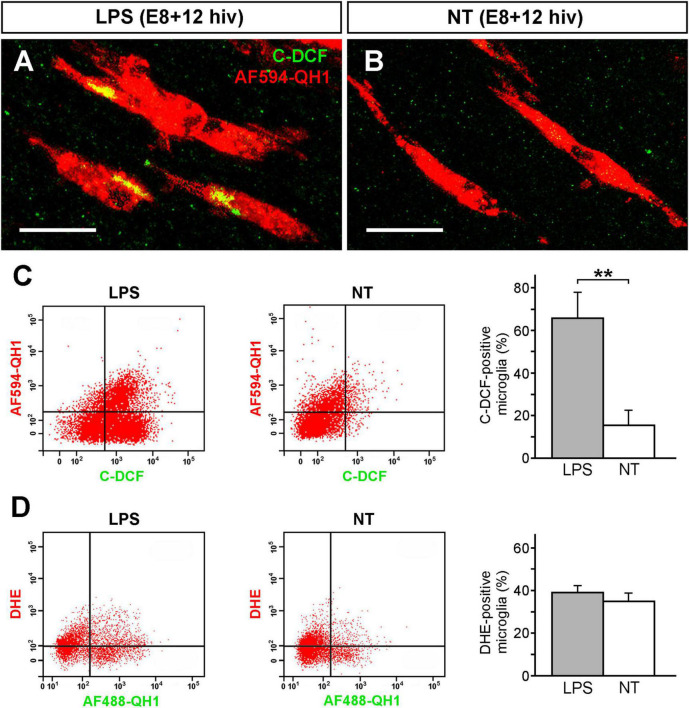
Increase of microglial reactive oxygen/nitrogen species (RONS) after LPS treatment. Microglial cells increase production of RONS but not superoxide (O_2_^–^) anions after LPS treatment of retina explants from E8 quail embryos cultured for 12 h *in vitro* (E8 + 12 hiv). **(A,B)** Representative confocal images of microglial cells from LPS-treated [LPS; **(A)**] and non-treated [NT; **(B)**] whole-mounted E8 + 12 hiv retina explants that were incubated for the last hiv in medium containing carboxy-H_2_DCFDA, which is converted to the fluorescent form carboxy-DCF (C-DCF, green) in the presence of RONS, and Alexa Fluor-594-conjugated QH1 antibody (AF594-QH1, red) as microglial marker. Images are maximum-intensity projections from Z stacks of confocal optical sections throughout the thickness of the ganglion cell layer (GCL). Scale bars: 20 μm. **(C)** Representative dot plots of flow cytometry analysis to study ROS production by microglial cells in LPS-treated (LPS, left) and non-treated (NT, right) E8 + 12 hiv retina explants. Carboxy-H_2_DCFDA and AF594-QH1 were added to the explant incubation medium for the last hiv. RONS-producing microglial cells (QH1- and C-DCF-positive cells) in the upper right quadrant of each dot plot are more abundant in LPS-treated versus non-treated explants. Bar diagram on the right displays the percentages (means ± SEM) of C-DCF-positive microglial cells from the whole population of microglia obtained in flow cytometry analysis of LPS-treated (grey bar) and non-treated (white bar) explants (*n* = 12 for each condition). The percentage of RONS-producing microglia is significantly higher in LPS-treated than in non-treated explants (**P < 0.01, Student’s t-test). **(D)** Representative dot plots of flow cytometry analysis to study O_2_^–^ anion production by microglial cells in LPS-treated (LPS, left) and non-treated (NT, right) E8 + 12 hiv retina explants after addition of dihydroethidium (DHE) and Alexa Fluor-488-conjugated QH1 antibody (AF488-QH1) to the incubation medium for the last hiv. Percentages (means ± SEM) of microglial cells producing O_2_^–^ anions (QH1- and DHE-positive cells) from the whole microglial population are shown in the bar diagram on the right. No significant differences (Student’s t-test) are found between LPS-treated (grey bar) and non-treated (white bar) explants (*n* = 6 for each condition), showing that O_2_^–^ anion production by microglia is not increased after LPS treatment of explants.

Generation of intracellular RONS was analyzed by using 6-carboxy-2′,7′-dichlorodihydrofluorescein diacetate (carboxy-H_2_DCFDA), a general oxidative stress indicator that detects the intracellular production of several RONS, excluding superoxide anions, by its conversion to the fluorescent form carboxy-DCF (C-DCF). Confocal microscopy analysis of intracellular RONS was performed on whole-mounted retina explants that were double-labeled with carboxy-H_2_DCFDA and the microglial marker Alexa Fluor 594-conjugated QH1 antibody (AF594-QH1). C-DCF-positive microglial cells were apparently more abundant in LPS-treated E8 + 12 hiv QEREs than in the non-treated controls (Figures 5A, B). C-DCF-positive microglial cells were quantified by flow cytometry on cell suspensions prepared from the double-labeled retina explants (Figure 5C). The percentage of microglial cells producing intracellular RONS was significantly higher in LPS-treated E8 + 12 hiv QEREs (65.8% ± 12.2) than in non-treated ones (15.4% ± 7.1) (Figure 5C).

The production of superoxide anions by microglial cells was detected by dihydroethidium (DHE) and the Alexa Fluor 488-conjugated QH1 antibody (AF488-QH1) in flow cytometry analysis. Our results revealed that the percentage of microglial cells producing superoxide anions (DHE-positive) did not significantly differ between LPS-treated and non-treated E8 + 12 hiv QEREs (Figure 5D). Thus, LPS treatment did not increase the production of superoxide anions. Despite that, it is noteworthy that more than 35% of microglial cells were DHE-positive in LPS-treated and non-treated explants, indicating that a considerable number of microglial cells produced superoxide anions independently of their functional state.

In summary, carboxy-H_2_DCFDA and DHE measurements demonstrated that LPS-treatment enhanced RONS but not superoxide anions production in microglia in E8 + 12 hiv QEREs. This is compatible with our previous work with fluorescent probe DAR-4M AM in E8 + 12 hiv QEREs showing an increased production of NO by LPS-stimulated microglia as well as the upregulation of iNOS expression at the protein and mRNA levels (Sierra et al., 2014). Hence, it can be concluded that NO was the main RONS produced by microglial cells after LPS treatment of E8 + 12 hiv QEREs.

### Inhibition of iNOS rescues a significant number of ganglion cells from death in LPS-treated QEREs

**FIGURE 6:**
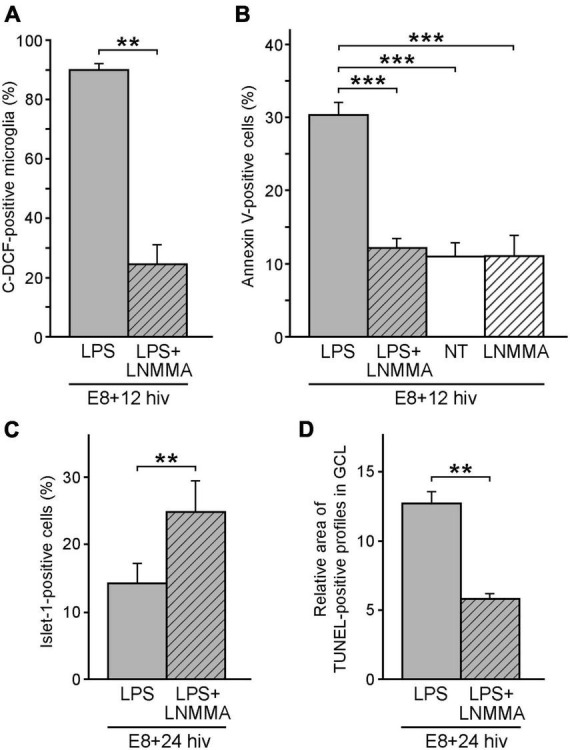
Reduction of microglial reactive oxygen/nitrogen species (RONS) and cell death by iNOS inhibition. iNOS inhibition by L-NMMA greatly decreases both microglial production of RONS **(A)** and cell death **(B,D)** in LPS-treated retina explants. It also increases the percentage of Islet 1-positive cells **(C)**. **(A)** Quantification of the flow cytometry analysis of RONS production by microglia in LPS-treated (LPS, grey bar) versus LPS plus L-NMMA-double treated (LPS + LNMMA, striped grey bar) retina explants from E8 quail embryos cultured for 12 h *in vitro* (E8 + 12 hiv). Bars represent the percentages (means ± SEM) of microglial cells (QH1-positive) producing ROS (C-DCF-positive) from the whole microglia population. The percentage of RONS-producing microglial cells is significantly higher in LPS-treated than in LPS + LNMMA explants (**P < 0.01, Student’s t-test; *n* = 6 for each condition). **(B)** Percentages (means ± SEM) of annexin V-positive cells in E8 + 12 hiv retina explants as determined by flow cytometry analysis in LPS-treated (LPS, grey bar), LPS plus L-NMMA-double-treated (LPS + LNMMA, striped grey bar), non-treated (NT, white bar), and L-NMMA-treated (LNMMA, striped white bar) explants (*n* = 12 for LPS and NT and *n* = 6 for LPS + LNMMA and LNMMA conditions). The percentage of annexin V-positive cells are significantly higher in LPS-treated explants, while the differences between LPS plus L-NMMA-double treated, non-treated and L-NMMA-treated explants are not significant (***P < 0.001, one-way ANOVA followed by Tukey test for multiple comparisons). **(C)** Percentages (means ± SEM) of Islet 1-positive cells as determined by flow cytometry analysis in LPS-treated (LPS, grey bar) and LPS plus L-NMMA-double treated (LPS + LNMMA, striped grey bar) E8 + 24 hiv retina explants (*n* = 11 for each condition). The percentage of Islet 1-positive cells is significantly lower in LPS-treated than LPS plus L-NMMA-double treated explants (**P < 0.01, Student’s t-test). **(D)** Relative areas of TUNEL-positive profiles in the ganglion cell layer (GCL) of E8 + 24 hiv retina explants as determined in two experiments comparing LPS-treated explants (LPS, grey bars) versus LPS plus L-NMMA double-treated (LPS + LNMMA, striped grey bar) explants. Data are presented as means ± SEM of percentages of TUNEL-positive pixels as measured in three microscopic fields of 250 μm × 250 μm in the ganglion cell layer (GCL) of whole-mounted explants (*n* = 8 for each condition). The relative area of TUNEL-positive profiles is significantly higher in LPS-treated than in LPS plus L-NMMA double-treated explants (**P < 0.01, Student’s t-test).

The above-described results demonstrated a significant reduction in the ganglion cell number after LPS treatment of E8 + 24 hiv QEREs, together with higher engagement of microglia in establishing phagocytic contacts with dying ganglion cells and increased microglial NO production after LPS-stimulation. This data strongly suggested that effective death of ganglion cells would be triggered by a microglia-promoted cell death with NO participation. To assess this hypothesis, we inhibited NO production by treating retinal explants with the iNOS inhibitor NG-monomethyl-L-arginine monoacetate (L-NMMA) and LPS (Figure 6). Cell suspensions from E8 + 12 hiv treated QEREs were stained with carboxy-H_2_DCFDA and AF594-QH1 and analyzed by flow cytometry. The percentage of microglial cells (QH1-positive) that produced RONS (C-DCF-positive) was significantly lower in LPS + L-NMMA-treated explants (24.5% ± 6.5) than in LPS-treated (89.9% ± 2.2) (Figure 6A). This revealed that L-NMMA effectively reduced RONS production by LPS-stimulated microglia.

The effect of L-NMMA treatment on apoptosis of retinal cell death (identified by the presence of externalized phosphatidylserine) was subsequently examined. Flow cytometry analysis was performed on cell suspensions from L-NMMA + LPS-treated and LPS-treated E8 + 12 hiv QEREs labeled with EGFP-annexin V (Figure 6B). The percentage of annexin V-positive cells significantly dropped from 30.3% (± 1.7) in LPS-treated explants to 12.2% (± 1.2) in LPS + L-NMMA-treated ones. In addition, no differences in the percentages of annexin V-positive cells were found between L-NMMA-treated and non-treated E8 + 12 hiv QEREs, thus showing that L-NMMA had no effect on the retinal cell death without LPS-stimulation. Altogether, experiments with annexin V demonstrated that L-NMMA reverted the effect of LPS, rescuing a similar number of cells from the LPS-induced phosphatidylserine externalization. This finding supports the idea that NO production by LPS-stimulated microglia might be the triggering signal for cell death in the retina at this developmental stage.

Since the population of Islet 1-positive cells (including ganglion cells) decreased in E8 + 24 hiv QEREs after LPS treatment (Figure 3B), simultaneous treatment with LPS and L-NMMA was performed on retina explants to test whether L-NMMA reverted the LPS-induced drop in the number of Islet 1-positive cells (Figure 6C). Flow cytometry analysis on cell suspensions from LPS + L-NMMA-treated and LPS-treated E8 + 24 hiv QEREs revealed a significant increase in the percentage of Islet 1-positive cells from 14.2% (± 2.9) in LPS-treated explants to 24.8% (± 4.5) in LPS + L-NMMA-treated ones (Figure 6C). Considering that ganglion cells are a subpopulation of Islet 1-positive retinal cells, we tested whether L-NMMA also had a positive effect on the survival of the ganglion cell population (Figure 6D). Hence, TUNEL-positive profiles in the GCL were compared between LPS + L-NMMA-treated and LPS-treated E8 + 24 hiv QEREs. The relative area occupied by TUNEL-positive profiles in the GCL (which is proportional to the number of dead ganglion cells) was significantly lower in LPS + L-NMMA-treated explants (5.8% ± 0.4) than in LPS-treated explants (12.7% ± 0.8) and similar to that found in non-treated explants (compare with Figure 2H). Therefore, we concluded that the fraction of the ganglion cell population induced to die by LPS treatment in E8 + 24 hiv QEREs was rescued from death after inhibiting the NO production by LPS-stimulated microglia. These results strongly supported that LPS-stimulated microglia promoted the death of ganglion cells through a NO-dependent mechanism.

## Discussion

Previous results in our laboratory showed that immature microglia increase the expression of the inflammatory markers iNOS and the production of NO during the development of quail embryo retina (Sierra et al., 2014). *In vitro* LPS treatment of QEREs modifies this basal phenotype of microglial cells in terms of additional changes in morphology, increased lysosomal compartment size, up-regulation of iNOS expression and the concomitant increased NO production (Figure 1; Sierra et al., 2014). In the present study, we demonstrate that LPS treatment of QEREs induces a decline in the ganglion cell population coinciding with an increase in the frequency of phagocytic contacts between microglial and dying ganglion cells. These findings support that the LPS-stimulation of immature microglia promotes ganglion cell death and are compatible with the existence of a mechanism of EPCD although a phagocytosis-independent mechanism cannot be ruled out. In addition, LPS treatment increases the production of RONS from microglia, including NO, but not affecting the production of superoxide anions. Importantly, we identified NO as key player involved in this LPS-induced microglial promotion of ganglion cell death, since iNOS inhibition by L-NMMA decreases the number of dead ganglion cells and increases the number of viable ganglion cells in LPS-treated retina explants.

Immature microglia in the normal developing CNS have specific traits, which are different from the ones of adult microglia (Matcovitch-Natan et al., 2016), showing mostly an amoeboid morphology with high migratory and phagocytic activities (Ling and Wong, 1993; Cuadros and Navascues, 1998; Marín-Teva et al., 1998, 1999; Rigato et al., 2011). We have used E8 QEREs as a model system to analyze if LPS-stimulated microglia can promote developmental ganglion cell death because this *in vitro*-system reproduces a physiological-like behavior of microglial cells similar to that seen in the developing retina *in situ* (Carrasco et al., 2011; Sierra et al., 2014). The term “microglial activation” has traditionally been referred to a process where mature microglia react against a damage or pathology in the CNS, which is characterized by a morphological transition from a ramified to a rounded or amoeboid phenotype, in addition to an increased migratory and phagocytic activity and a higher release of a great variety of molecules including cytotoxic factors, such as NO (Garden and Möller, 2006; Hanisch and Kettenmann, 2007; Boche et al., 2013). Nevertheless, we consider that immature microglia in the developing CNS might show a certain degree of upregulation of these damage-related responses that we named “basal phenotype.” Thus, immature microglia in the developing CNS and traditionally “activated” microglia in the injured adult CNS share iNOS expression (Crain et al., 2013; Cunningham et al., 2013; Sierra et al., 2014), being one of the main microglial inflammatory markers (Bal-Price and Brown, 2001; Garden and Möller, 2006).

The fact that iNOS inhibition rescued a significant number of ganglion cells from death in LPS-treated retina explants is in line with previous studies showing cell death promoted by phagocytosis in co-cultures of neurons and microglia stimulated with LPS, β-amyloid 1-42, lipoteichoic acid or tumor necrosis factor-α (Neher et al., 2011, 2014; Neniskyte et al., 2011, 2014, 2016; Fricker et al., 2012a,b). In all of these studies iNOS expression and production of NO, which can react with superoxide anions to form peroxynitrite, are elevated. In this case, peroxynitrite produced by microglia would induce a reversible externalization of phosphatidylserine in neurons that are going to be eliminated through an EPCD mechanism. Alternatively, NO, without binding to the superoxide, might be sufficient to induce a mild and reversible caspase-3 activation causing reversible phosphatidylserine exposure and/or the EPCD process, as demonstrated in several *in vitro* studies (Hornik et al., 2016; Brelstaff et al., 2018). It has also been observed that LPS and IFN-γ-stimulated microglia induce neuronal death through NO-production, requiring direct cell-to-cell contacts between microglia and degenerating neurons (Gibbons and Dragunow, 2006). This is supported by the findings that NO produced by iNOS increases the phagocytic capacity of microglia (Maksoud et al., 2019).

A similar picture might occur in QEREs after LPS treatment, where the increase in cells with externalized phosphatidylserine coincides with the rise in the proportion of NO-producing microglial cells and that of phagocytic contacts between microglial cells and caspase-3-positive ganglion cells. The superoxide anion was already present before treating microglia with LPS, and therefore an increase in NO would induce (alone or in combination with superoxide to form peroxynitrite) phosphatidylserine exposure resulting in the increase of EPCD of ganglion cells in cultured QEREs. However, our results do not unequivocally demonstrate the existence of an EPCD mechanism and therefore an engulfment-independent mechanism of cell death induction cannot be excluded.

Furthermore, the addition of the iNOS inhibitor L-NMMA to E8 QEREs reduces the production of NO by microglial cells, phosphatidylserine externalization, and TUNEL-positive profiles in the GCL, together with the rescue of ganglion cells, supporting the hypothesis that LPS-stimulated microglia induce ganglion cell death. Comparable reductions in cell death induced by inflammatory microglia has been demonstrated after L-NMMA or other iNOS inhibitors treatment in mixed neuronal and microglia co-cultures from mouse developing brain pretreated with LPS and IFN-γ (Chao et al., 1992), and in co-cultures of oligodendrocytes and microglia or neurons and microglia from postnatal rat cerebral cortex or cerebellum treated with LPS, with LPS and INFγ, or with β-amyloid 1-42 peptide (Xie et al., 2002; Li et al., 2005; Neher et al., 2011; Hornik et al., 2016; Brelstaff et al., 2018).

Thus, the present study only provides evidence for the existence of ganglion cell death induced by LPS-stimulated microglia in E8 QEREs, representing a mechanism which could also operate during the *in situ* development of the quail retina. Four lines of evidence support this notion. First, there is a chronological coincidence between naturally occurring death of ganglion cells and microglial colonization of the GCL in the *in situ* developing retina (Navascués et al., 1995; Marín-Teva et al., 1999). In fact, at E8, which is the developmental age when QEREs were collected for this study, microglial cells are tangentially migrating on the nerve fiber layer of the quail retina, reaching the GCL where programmed death of numerous ganglion cells takes place. Second, LPS-stimulation of E8 QERES without microglia did not increase phosphatidylserine externalization in retinal cells, demonstrating that this process is mediated by microglia (see Figure 2). Third, numerous phagocytic contacts between microglia and caspase-3-positive ganglion cells are also detected in *in situ* quail embryo retinas at E8, as was found in both control and LPS-treated E8 QEREs (see Figure 4). And fourth, microglia in the nerve fiber layer and GCL of *in situ* quail embryo retinas show a strong iNOS immunolabeling with concomitant production of NO (Sierra et al., 2014), which could presumably promote neighboring-ganglion cell death. In addition, neuronal cell death induced by microglia appears to be a generalized mechanism not only during the development of the retina but also in response to retinal pathology and injury. For example, phagocytic engulfment of non-apoptotic rods by microglia has been demonstrated to contribute to photoreceptor death in the process of retinal degeneration in mice with retinitis pigmentosa (Zhao et al., 2015; Zabel et al., 2016).

In summary, our results demonstrate an increment in ganglion cell death carried out by LPS-stimulated microglia, by an NO- and/or its derivative peroxinytrite-dependent mechanism. Our data suggest that this microglial induction of ganglion cell death might also take place during quail retinal development, but further studies are needed to prove this hypothesis.

## Data availability statement

The original contributions presented in this study are included in the article/supplementary material, further inquiries can be directed to the corresponding author.

## Ethics statement

This animal study was reviewed and approved by Animal Experimentation Ethics Committee of the University of Granada, following the guidelines of the European Union Directive 2010/63/EU on the protection of animals used for scientific purposes.

## Author contributions

AS-M, JN, MC, and JM-T contributed to the conception and design of the study. AS-M, VN, MS, and DM-O performed the experiments and the statistical analysis. AS-M, JN, and JM-T wrote the first draft of the manuscript. VN, MS, and MC wrote sections of the manuscript. All authors contributed to manuscript revision, read, and approved the submitted version.
